# Crystal structure of ethyl (4*R*)-2-amino-7-hy­droxy-4-phenyl-4*H*-chromene-3-carboxyl­ate

**DOI:** 10.1107/S2056989015012013

**Published:** 2015-06-27

**Authors:** Joel T. Mague, Shaaban K. Mohamed, Mehmet Akkurt, Sabry H. H. Younes, Mustafa R. Albayati

**Affiliations:** a Department of Chemistry, Tulane University, New Orleans, LA 70118, USA; b Faculty of Science & Engineering, School of Healthcare Science, Manchester Metropolitan University, Manchester M1 5GD, England; c Chemistry Department, Faculty of Science, Minia University, 61519 El-Minia, Egypt; d Department of Physics, Faculty of Sciences, Erciyes University, 38039 Kayseri, Turkey; e Chemistry Department, Faculty of Science, Sohag University, 82524 Sohag, Egypt; f Kirkuk University, College of Education, Department of Chemistry, Kirkuk, Iraq

**Keywords:** crystal structure, amino chromenes, 4*H*-chromene, hydrogen bonding

## Abstract

In the title compound, C_18_H_17_NO_4_, the dihedral angle between the phenyl ring and the fused six-membered ring is 77.65 (4)°. The conformation of the mol­ecule is determined in part by an intra­molecular N—H⋯O hydrogen bond between the amino H atom and the carbonyl O atom, forming an *S*(6) motif. In the crystal, mol­ecules are linked into N—H⋯O hydrogen-bonded inversion dimers which are then connected into chains along [001], forming a two-dimensional network parallel to (100) *via* O—H⋯O hydrogen bonds. C—H⋯O interactions further contribute to the crystal stability. The ethyl group is disordered over two sets of sites in a 0.801 (5):0.199 (5) ratio.

## Related literature   

For background to the synthesis and biological activity of mol­ecules having a 4*H*-chromene or 4*H*-benzochromene residue, see: Kiyani & Ghorbani (2014 ▸); Kale *et al.* (2013 ▸); Sabry *et al.* (2011 ▸); Kidwai *et al.* (2010 ▸); Mungra *et al.* (2011 ▸); Cingolani *et al.* (1969 ▸); Wu *et al.* (2003 ▸); Perrella *et al.* (1994 ▸); Patil *et al.* (1993 ▸); Emmadi *et al.* (2012 ▸); Wang *et al.* (2003 ▸); Armesto *et al.* (1989 ▸).
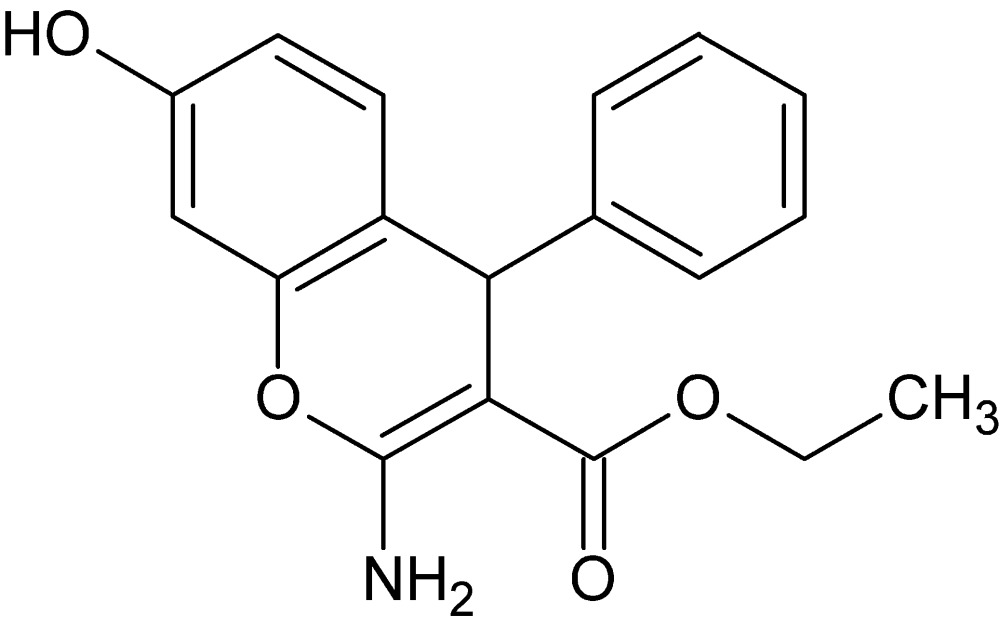


## Experimental   

### Crystal data  

C_18_H_17_NO_4_
*M* *_r_* = 311.32
Monoclinic, 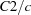

*a* = 31.5071 (7) Å
*b* = 5.8582 (1) Å
*c* = 21.2249 (5) Å
β = 130.180 (1)°
*V* = 2993.11 (11) Å^3^
*Z* = 8
Cu *K*α radiation
μ = 0.81 mm^−1^
*T* = 150 K
0.22 × 0.18 × 0.02 mm

### Data collection  

Bruker D8 VENTURE PHOTON 100 CMOS diffractometer
Absorption correction: multi-scan (*SADABS*; Bruker, 2014 ▸) *T*_min_ = 0.91, *T*_max_ = 0.98
11241 measured reflections
2891 independent reflections
2348 reflections with *I* > 2σ(*I*)
*R*_int_ = 0.033

### Refinement  

*R*[*F* ^2^ > 2σ(*F* ^2^)] = 0.038
*wR*(*F* ^2^) = 0.101
*S* = 1.05
2891 reflections
227 parameters
2 restraints
H atoms treated by a mixture of independent and constrained refinement
Δρ_max_ = 0.27 e Å^−3^
Δρ_min_ = −0.26 e Å^−3^

### 

Data collection: *APEX2* (Bruker, 2014 ▸); cell refinement: *SAINT* (Bruker, 2014 ▸); data reduction: *SAINT*; program(s) used to solve structure: *SHELXT* (Sheldrick, 2015*a* ▸); program(s) used to refine structure: *SHELXL2014* (Sheldrick, 2015*b* ▸); molecular graphics: *DIAMOND* (Brandenburg & Putz, 2012 ▸); software used to prepare material for publication: *SHELXTL* (Sheldrick, 2008 ▸).

## Supplementary Material

Crystal structure: contains datablock(s) global, I. DOI: 10.1107/S2056989015012013/qm2111sup1.cif

Structure factors: contains datablock(s) I. DOI: 10.1107/S2056989015012013/qm2111Isup2.hkl

Supporting information file. DOI: 10.1107/S2056989015012013/qm2111Isup3.cml

**Fig. 1.**
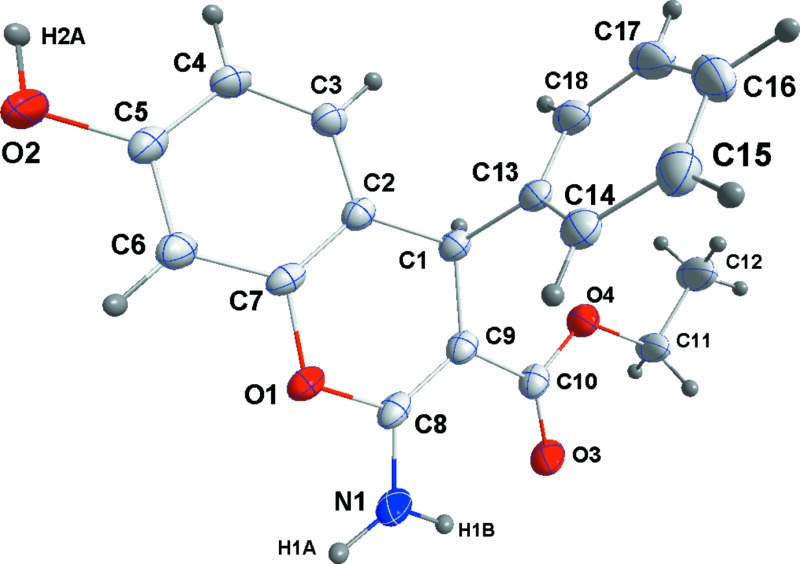
The title molecule with labeling scheme and 50% probability ellipsoids. Only one orientation of the disordered ethyl group is shown.

**Fig. 2.**
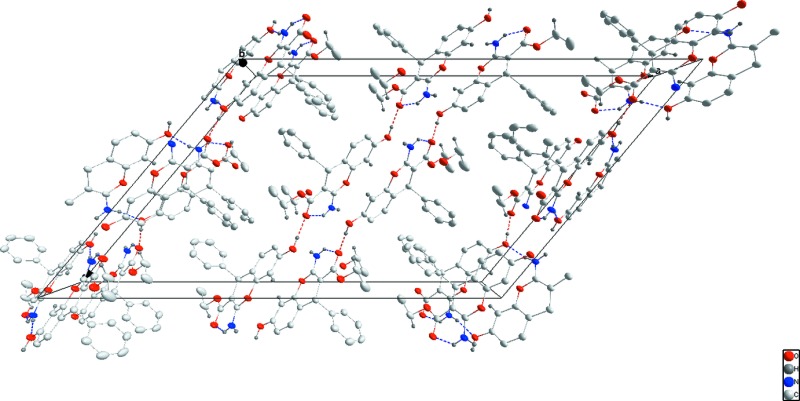
Packing viewed down the b axis. N—H···O and O—H···O hydrogen bonds are shown, respectively as blue and red dotted lines.

. DOI: 10.1107/S2056989015012013/qm2111fig1.tif
The title mol­ecule with labeling scheme and 50% probability ellipsoids. Only one orientation of the disordered ethyl group is shown.

b . DOI: 10.1107/S2056989015012013/qm2111fig2.tif
Packing viewed down the *b* axis. N—H⋯O and O—H⋯O hydrogen bonds are shown, respectively as blue and red dotted lines.

CCDC reference: 1408238

Additional supporting information:  crystallographic information; 3D view; checkCIF report

## Figures and Tables

**Table 1:** Hydrogen-bond geometry (, )

*D*H*A*	*D*H	H*A*	*D* *A*	*D*H*A*
C11H11*A*O2^i^	0.99	2.58	3.312(3)	131
C6H6O1^ii^	0.95	2.56	3.4736(17)	163
N1H1*B*O3	0.88(2)	1.998(19)	2.6840(18)	133.7(16)
N1H1*A*O2^ii^	0.93(2)	2.15(2)	3.0710(18)	169.6(17)
O2H2*A*O3^iii^	0.90(2)	1.83(2)	2.7331(15)	179(2)

Symmetry codes: (i) 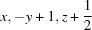; (ii) 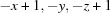; (iii) 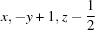.

## supplementary crystallographic information

### S1. Comment

Besides the various biological properties of 2-Amino-4*H*-Chromenes, they also act as important synthetic building blocks for various bio-active molecules (Kiyani & Ghorbani, 2014; Kale *et al.*, 2013; Sabry *et al*., 2011; Kidwai *et al.*, 2010). During the last decade, such compounds had shown interesting pharmacological properties such as antimicrobial and anti-tuberculosis agents (Mungra *et al.*, 2011), anticoagulant (Cingolani *et al.*, 1969), anticancer (Wu *et al.*, 2003), antitumour (Perrella, *et al.*, 1994), cytotoxic and anti-HIV activities (Patil *et al.*, 1993; Emmadi, *et al.*, 2012). Also, chromenes are also structural features of various natural products (Wang *et al*., 2003) and possess useful photochemical properties (Armesto *et al*., 1989).

In the title molecule, the dihedral angle between the phenyl ring (C13–C18) and the C2–C7 ring is 77.65 (4)°. A puckering analysis of the heterocyclic ring gave Q = 0.118 (2) Å, θ = 95.1 (8)° and φ = 352.0 (8)°. The conformation of the molecule is determined in part by an intramolecular N1—H1B···O3 hydrogen bond. Pairwise N1—H1A···O2^i^ (i: 1 - *x*, -*y*, 1 - *z*) hydrogen bonds form dimers which are then connected into chains *via* O2—H2A···O3^ii^ (ii: *x*, 1 - *y*, -1/2 + *z*) hydrogen bonds (Table 1 and Fig. 2).

### S2. Experimental

The title compound was synthesized by the reaction of (*E*)-ethyl 3-(phenyl)-2-cyanoacrylate (1 mmol, 201 mg) and 1,3-Benzenediol (1 mmol, 110 mg) catalyzed by Et_3_N in 10 ml e thanol at the refuxing temperature. After cooling, the solvent was removed under reduced pressure and the residue was washed with cold ethanol and recrystallized from ethanol to afford pure colourless crystals suitable for X-ray diffraction in 92% yeild and *M*.p 491 K.

### S3. Refinement

H-atoms were placed in calculated positions (C—H = 0.95 - 0.98 Å) and included as riding contributions with isotropic displacement parameters 1.2 - 1.5 times those of the attached carbon atoms. The ethyl group (C11,C12) is disordered over two sites. The components of the disorder were refined subject to restraints that their geometries be approximately the same.

### Crystal data

**Table:**

C_18_H_17_NO_4_	*F*(000) = 1312
*M**_r_* = 311.32	*D*_x_ = 1.382 Mg m^−^^3^
Monoclinic, *C*2/*c*	Cu *K*α radiation, λ = 1.54178 Å
*a* = 31.5071 (7) Å	Cell parameters from 6704 reflections
*b* = 5.8582 (1) Å	θ = 3.7–72.2°
*c* = 21.2249 (5) Å	µ = 0.81 mm^−^^1^
β = 130.180 (1)°	*T* = 150 K
*V* = 2993.11 (11) Å^3^	Plate, colourless
*Z* = 8	0.22 × 0.18 × 0.02 mm

### Data collection

**Table:**

Bruker D8 VENTURE PHOTON 100 CMOS diffractometer	2891 independent reflections
Radiation source: INCOATEC IµS micro–focus source	2348 reflections with *I* > 2σ(*I*)
Mirror monochromator	*R*_int_ = 0.033
ω scans	θ_max_ = 72.2°, θ_min_ = 3.7°
Absorption correction: multi-scan (*SADABS*; Bruker, 2014)	*h* = −38→36
*T*_min_ = 0.91, *T*_max_ = 0.98	*k* = −6→7
11241 measured reflections	*l* = −24→26

### Refinement

**Table:**

Refinement on *F*^2^	Primary atom site location: structure-invariant direct methods
Least-squares matrix: full	Secondary atom site location: difference Fourier map
*R*[*F*^2^ > 2σ(*F*^2^)] = 0.038	Hydrogen site location: mixed
*wR*(*F*^2^) = 0.101	H atoms treated by a mixture of independent and constrained refinement
*S* = 1.05	*w* = 1/[σ^2^(*F*_o_^2^) + (0.049*P*)^2^ + 1.7699*P*] where *P* = (*F*_o_^2^ + 2*F*_c_^2^)/3
2891 reflections	(Δ/σ)_max_ < 0.001
227 parameters	Δρ_max_ = 0.27 e Å^−^^3^
2 restraints	Δρ_min_ = −0.25 e Å^−^^3^

### Special details

**Table:**

Geometry. All e.s.d.'s (except the e.s.d. in the dihedral angle between two l.s. planes) are estimated using the full covariance matrix. The cell e.s.d.'s are taken into account individually in the estimation of e.s.d.'s in distances, angles and torsion angles; correlations between e.s.d.'s in cell parameters are only used when they are defined by crystal symmetry. An approximate (isotropic) treatment of cell e.s.d.'s is used for estimating e.s.d.'s involving l.s. planes.
Refinement. Refinement of *F*^2^ against ALL reflections. The weighted *R*-factor *wR* and goodness of fit *S* are based on *F*^2^, conventional *R*-factors *R* are based on *F*, with *F* set to zero for negative *F*^2^. The threshold expression of *F*^2^ > σ(*F*^2^) is used only for calculating *R*-factors(gt) *etc*. and is not relevant to the choice of reflections for refinement. *R*-factors based on *F*^2^ are statistically about twice as large as those based on *F*, and *R*- factors based on ALL data will be even larger. H-atoms were placed in calculated positions (C—H = 0.95 - 0.98 Å) and included as riding contributions with isotropic displacement parameters 1.2 - 1.5 times those of the attached carbon atoms. The ethyl group (C11,C12) is disordered over two sites. The components of the disorder were refined subject to restraints that their geometries be approximately the same.

### Fractional atomic coordinates and isotropic or equivalent isotropic displacement parameters (Å^2^)

**Table:**

	*x*	*y*	*z*	*U*_iso_*/*U*_eq_	Occ. (<1)
O1	0.45362 (4)	0.22755 (17)	0.51953 (6)	0.0270 (2)	
O2	0.46127 (4)	0.21959 (19)	0.30568 (6)	0.0302 (3)	
H2A	0.4494 (9)	0.294 (4)	0.2598 (14)	0.056 (6)*	
O3	0.42602 (4)	0.5543 (2)	0.66661 (6)	0.0340 (3)	
O4	0.37668 (5)	0.82032 (19)	0.56697 (6)	0.0338 (3)	
N1	0.46907 (5)	0.2182 (3)	0.63712 (8)	0.0305 (3)	
H1A	0.4890 (8)	0.084 (4)	0.6479 (12)	0.049 (5)*	
H1B	0.4647 (8)	0.277 (3)	0.6709 (12)	0.039 (5)*	
C1	0.38998 (6)	0.6527 (2)	0.46364 (8)	0.0242 (3)	
H1	0.4037	0.8139	0.4768	0.029*	
C2	0.40953 (5)	0.5419 (2)	0.42173 (8)	0.0228 (3)	
C3	0.39849 (6)	0.6388 (3)	0.35231 (8)	0.0256 (3)	
H3	0.3789	0.7795	0.3318	0.031*	
C4	0.41512 (6)	0.5365 (3)	0.31246 (8)	0.0260 (3)	
H4	0.4070	0.6063	0.2654	0.031*	
C5	0.44383 (6)	0.3298 (2)	0.34225 (8)	0.0241 (3)	
C6	0.45583 (6)	0.2297 (2)	0.41125 (8)	0.0243 (3)	
H6	0.4754	0.0892	0.4320	0.029*	
C7	0.43866 (5)	0.3386 (2)	0.44957 (8)	0.0227 (3)	
C8	0.44500 (6)	0.3350 (3)	0.56735 (8)	0.0244 (3)	
C9	0.41527 (5)	0.5333 (3)	0.54438 (8)	0.0248 (3)	
C10	0.40769 (6)	0.6307 (3)	0.59859 (9)	0.0274 (3)	
C11	0.35861 (16)	0.9101 (9)	0.60985 (17)	0.0450 (8)	0.801 (5)
H11A	0.3904	0.9738	0.6641	0.054*	0.801 (5)
H11B	0.3409	0.7892	0.6185	0.054*	0.801 (5)
C12	0.31703 (14)	1.0965 (6)	0.55374 (19)	0.0619 (10)	0.801 (5)
H12A	0.3029	1.1656	0.5791	0.093*	0.801 (5)
H12B	0.3353	1.2137	0.5456	0.093*	0.801 (5)
H12C	0.2861	1.0303	0.5003	0.093*	0.801 (5)
C11A	0.3518 (6)	0.940 (5)	0.5958 (8)	0.0450 (8)	0.199 (5)
H11C	0.3677	1.0951	0.6149	0.054*	0.199 (5)
H11D	0.3586	0.8561	0.6421	0.054*	0.199 (5)
C12A	0.2899 (5)	0.953 (3)	0.5230 (7)	0.0619 (10)	0.199 (5)
H12D	0.2712	1.0330	0.5397	0.093*	0.199 (5)
H12E	0.2838	1.0361	0.4777	0.093*	0.199 (5)
H12F	0.2748	0.7983	0.5047	0.093*	0.199 (5)
C13	0.32653 (6)	0.6603 (2)	0.40441 (8)	0.0248 (3)	
C14	0.29555 (6)	0.4724 (3)	0.39345 (9)	0.0289 (3)	
H14	0.3139	0.3368	0.4240	0.035*	
C15	0.23796 (6)	0.4812 (3)	0.33820 (10)	0.0369 (4)	
H15	0.2171	0.3520	0.3314	0.044*	
C16	0.21071 (7)	0.6774 (3)	0.29294 (10)	0.0415 (4)	
H16	0.1713	0.6836	0.2555	0.050*	
C17	0.24104 (7)	0.8635 (3)	0.30240 (10)	0.0399 (4)	
H17	0.2225	0.9975	0.2707	0.048*	
C18	0.29867 (7)	0.8557 (3)	0.35817 (9)	0.0328 (3)	
H18	0.3193	0.9854	0.3648	0.039*	

### Atomic displacement parameters (Å^2^)

**Table:**

	*U*^11^	*U*^22^	*U*^33^	*U*^12^	*U*^13^	*U*^23^
O1	0.0334 (5)	0.0318 (5)	0.0209 (5)	0.0037 (4)	0.0198 (4)	0.0024 (4)
O2	0.0346 (6)	0.0392 (6)	0.0237 (5)	0.0060 (5)	0.0220 (5)	0.0032 (5)
O3	0.0372 (6)	0.0470 (7)	0.0225 (5)	0.0043 (5)	0.0214 (5)	0.0005 (5)
O4	0.0401 (6)	0.0395 (6)	0.0287 (5)	0.0059 (5)	0.0254 (5)	−0.0007 (5)
N1	0.0331 (7)	0.0414 (8)	0.0214 (6)	0.0060 (6)	0.0196 (6)	0.0041 (6)
C1	0.0266 (7)	0.0270 (7)	0.0210 (7)	−0.0031 (6)	0.0163 (6)	−0.0034 (6)
C2	0.0209 (6)	0.0280 (7)	0.0191 (6)	−0.0036 (6)	0.0127 (5)	−0.0033 (6)
C3	0.0240 (7)	0.0293 (7)	0.0235 (7)	−0.0011 (6)	0.0153 (6)	0.0008 (6)
C4	0.0264 (7)	0.0328 (8)	0.0208 (7)	−0.0026 (6)	0.0161 (6)	0.0015 (6)
C5	0.0216 (6)	0.0334 (8)	0.0192 (6)	−0.0036 (6)	0.0140 (5)	−0.0044 (6)
C6	0.0227 (7)	0.0287 (7)	0.0202 (7)	0.0003 (6)	0.0134 (6)	0.0001 (6)
C7	0.0221 (6)	0.0297 (7)	0.0156 (6)	−0.0045 (5)	0.0118 (5)	−0.0011 (5)
C8	0.0221 (7)	0.0349 (8)	0.0174 (6)	−0.0044 (6)	0.0132 (6)	−0.0036 (6)
C9	0.0220 (7)	0.0334 (8)	0.0182 (6)	−0.0026 (6)	0.0127 (6)	−0.0031 (6)
C10	0.0241 (7)	0.0358 (8)	0.0225 (7)	−0.0031 (6)	0.0151 (6)	−0.0042 (6)
C11	0.0614 (14)	0.050 (2)	0.0456 (14)	0.0246 (12)	0.0448 (12)	0.0156 (16)
C12	0.079 (2)	0.074 (2)	0.0624 (18)	0.0394 (17)	0.0588 (18)	0.0292 (16)
C11A	0.0614 (14)	0.050 (2)	0.0456 (14)	0.0246 (12)	0.0448 (12)	0.0156 (16)
C12A	0.079 (2)	0.074 (2)	0.0624 (18)	0.0394 (17)	0.0588 (18)	0.0292 (16)
C13	0.0276 (7)	0.0305 (7)	0.0194 (6)	0.0023 (6)	0.0166 (6)	−0.0012 (6)
C14	0.0298 (7)	0.0344 (8)	0.0252 (7)	0.0014 (6)	0.0190 (6)	−0.0008 (6)
C15	0.0302 (8)	0.0528 (10)	0.0314 (8)	−0.0043 (7)	0.0216 (7)	−0.0079 (8)
C16	0.0257 (8)	0.0665 (12)	0.0272 (8)	0.0113 (8)	0.0148 (7)	−0.0040 (8)
C17	0.0418 (9)	0.0470 (10)	0.0285 (8)	0.0186 (8)	0.0216 (7)	0.0054 (7)
C18	0.0407 (9)	0.0333 (8)	0.0283 (8)	0.0068 (7)	0.0241 (7)	0.0021 (7)

### Geometric parameters (Å, º)

**Table:**

O1—C8	1.3622 (16)	C9—C10	1.4358 (19)
O1—C7	1.3954 (16)	C11—C12	1.521 (4)
O2—C5	1.3679 (17)	C11—H11A	0.9900
O2—H2A	0.90 (2)	C11—H11B	0.9900
O3—C10	1.2419 (18)	C12—H12A	0.9800
O4—C10	1.3387 (18)	C12—H12B	0.9800
O4—C11	1.448 (2)	C12—H12C	0.9800
O4—C11A	1.448 (4)	C11A—C12A	1.519 (6)
N1—C8	1.3366 (19)	C11A—H11C	0.9900
N1—H1A	0.93 (2)	C11A—H11D	0.9900
N1—H1B	0.88 (2)	C12A—H12D	0.9800
C1—C2	1.5164 (18)	C12A—H12E	0.9800
C1—C9	1.5165 (19)	C12A—H12F	0.9800
C1—C13	1.5284 (19)	C13—C14	1.388 (2)
C1—H1	1.0000	C13—C18	1.390 (2)
C2—C7	1.382 (2)	C14—C15	1.387 (2)
C2—C3	1.3976 (19)	C14—H14	0.9500
C3—C4	1.386 (2)	C15—C16	1.384 (2)
C3—H3	0.9500	C15—H15	0.9500
C4—C5	1.395 (2)	C16—C17	1.376 (3)
C4—H4	0.9500	C16—H16	0.9500
C5—C6	1.3846 (19)	C17—C18	1.388 (2)
C6—C7	1.3886 (19)	C17—H17	0.9500
C6—H6	0.9500	C18—H18	0.9500
C8—C9	1.369 (2)		
			
C8—O1—C7	118.99 (11)	O4—C11—H11A	110.8
C5—O2—H2A	110.5 (14)	C12—C11—H11A	110.8
C10—O4—C11	116.23 (19)	O4—C11—H11B	110.8
C10—O4—C11A	127.5 (10)	C12—C11—H11B	110.8
C8—N1—H1A	120.8 (12)	H11A—C11—H11B	108.8
C8—N1—H1B	115.2 (12)	C11—C12—H12A	109.5
H1A—N1—H1B	124.0 (17)	C11—C12—H12B	109.5
C2—C1—C9	110.43 (12)	H12A—C12—H12B	109.5
C2—C1—C13	109.92 (11)	C11—C12—H12C	109.5
C9—C1—C13	113.43 (11)	H12A—C12—H12C	109.5
C2—C1—H1	107.6	H12B—C12—H12C	109.5
C9—C1—H1	107.6	O4—C11A—C12A	106.5 (7)
C13—C1—H1	107.6	O4—C11A—H11C	110.4
C7—C2—C3	116.48 (12)	C12A—C11A—H11C	110.4
C7—C2—C1	121.82 (12)	O4—C11A—H11D	110.4
C3—C2—C1	121.69 (13)	C12A—C11A—H11D	110.4
C4—C3—C2	122.18 (14)	H11C—C11A—H11D	108.6
C4—C3—H3	118.9	C11A—C12A—H12D	109.5
C2—C3—H3	118.9	C11A—C12A—H12E	109.5
C3—C4—C5	119.26 (13)	H12D—C12A—H12E	109.5
C3—C4—H4	120.4	C11A—C12A—H12F	109.5
C5—C4—H4	120.4	H12D—C12A—H12F	109.5
O2—C5—C6	117.71 (13)	H12E—C12A—H12F	109.5
O2—C5—C4	122.20 (12)	C14—C13—C18	118.64 (14)
C6—C5—C4	120.09 (13)	C14—C13—C1	121.35 (13)
C5—C6—C7	118.79 (13)	C18—C13—C1	119.98 (14)
C5—C6—H6	120.6	C15—C14—C13	120.48 (15)
C7—C6—H6	120.6	C15—C14—H14	119.8
C2—C7—C6	123.19 (12)	C13—C14—H14	119.8
C2—C7—O1	122.14 (12)	C16—C15—C14	120.31 (16)
C6—C7—O1	114.67 (12)	C16—C15—H15	119.8
N1—C8—O1	110.23 (13)	C14—C15—H15	119.8
N1—C8—C9	126.67 (13)	C17—C16—C15	119.66 (15)
O1—C8—C9	123.09 (12)	C17—C16—H16	120.2
C8—C9—C10	118.78 (13)	C15—C16—H16	120.2
C8—C9—C1	122.26 (12)	C16—C17—C18	120.10 (16)
C10—C9—C1	118.96 (13)	C16—C17—H17	119.9
O3—C10—O4	121.56 (13)	C18—C17—H17	119.9
O3—C10—C9	126.52 (14)	C17—C18—C13	120.79 (16)
O4—C10—C9	111.92 (12)	C17—C18—H18	119.6
O4—C11—C12	105.0 (2)	C13—C18—H18	119.6
			
C9—C1—C2—C7	9.72 (18)	C13—C1—C9—C8	115.70 (15)
C13—C1—C2—C7	−116.18 (14)	C2—C1—C9—C10	171.99 (12)
C9—C1—C2—C3	−170.99 (12)	C13—C1—C9—C10	−64.11 (17)
C13—C1—C2—C3	63.11 (17)	C11—O4—C10—O3	−9.3 (3)
C7—C2—C3—C4	0.7 (2)	C11A—O4—C10—O3	−11.6 (12)
C1—C2—C3—C4	−178.58 (13)	C11—O4—C10—C9	169.8 (3)
C2—C3—C4—C5	0.0 (2)	C11A—O4—C10—C9	167.5 (12)
C3—C4—C5—O2	179.81 (13)	C8—C9—C10—O3	0.7 (2)
C3—C4—C5—C6	−0.5 (2)	C1—C9—C10—O3	−179.51 (14)
O2—C5—C6—C7	179.87 (12)	C8—C9—C10—O4	−178.39 (12)
C4—C5—C6—C7	0.1 (2)	C1—C9—C10—O4	1.43 (18)
C3—C2—C7—C6	−1.1 (2)	C10—O4—C11—C12	−170.7 (3)
C1—C2—C7—C6	178.23 (13)	C10—O4—C11A—C12A	−123.6 (13)
C3—C2—C7—O1	178.25 (12)	C2—C1—C13—C14	82.33 (16)
C1—C2—C7—O1	−2.4 (2)	C9—C1—C13—C14	−41.85 (18)
C5—C6—C7—C2	0.7 (2)	C2—C1—C13—C18	−95.55 (15)
C5—C6—C7—O1	−178.71 (12)	C9—C1—C13—C18	140.28 (13)
C8—O1—C7—C2	−7.73 (19)	C18—C13—C14—C15	−0.8 (2)
C8—O1—C7—C6	171.67 (11)	C1—C13—C14—C15	−178.73 (13)
C7—O1—C8—N1	−170.97 (11)	C13—C14—C15—C16	0.4 (2)
C7—O1—C8—C9	9.47 (19)	C14—C15—C16—C17	0.6 (2)
N1—C8—C9—C10	−0.6 (2)	C15—C16—C17—C18	−1.2 (2)
O1—C8—C9—C10	178.86 (12)	C16—C17—C18—C13	0.7 (2)
N1—C8—C9—C1	179.56 (13)	C14—C13—C18—C17	0.3 (2)
O1—C8—C9—C1	−1.0 (2)	C1—C13—C18—C17	178.21 (13)
C2—C1—C9—C8	−8.20 (18)		

### Hydrogen-bond geometry (Å, º)

**Table:**

*D*—H···*A*	*D*—H	H···*A*	*D*···*A*	*D*—H···*A*
C11—H11*A*···O2^i^	0.99	2.58	3.312 (3)	131
C6—H6···O1^ii^	0.95	2.56	3.4736 (17)	163
N1—H1*B*···O3	0.88 (2)	1.998 (19)	2.6840 (18)	133.7 (16)
N1—H1*A*···O2^ii^	0.93 (2)	2.15 (2)	3.0710 (18)	169.6 (17)
O2—H2*A*···O3^iii^	0.90 (2)	1.83 (2)	2.7331 (15)	179 (2)

Symmetry codes: (i) *x*, −*y*+1, *z*+1/2; (ii) −*x*+1, −*y*, −*z*+1; (iii) *x*, −*y*+1, *z*−1/2.
