# Supplementary figures and images for: Crystal structure of ethyl (4R)-2-amino-7-hy­droxy-4-phenyl-4H-chromene-3-carboxyl­ate
Source: Acta Crystallogr E Crystallogr Commun. 2015 Jun 27;71(Pt 7):o519–20. doi: 10.1107/S2056989015012013 (PMC4518989; doi:10.1107/S2056989015012013)

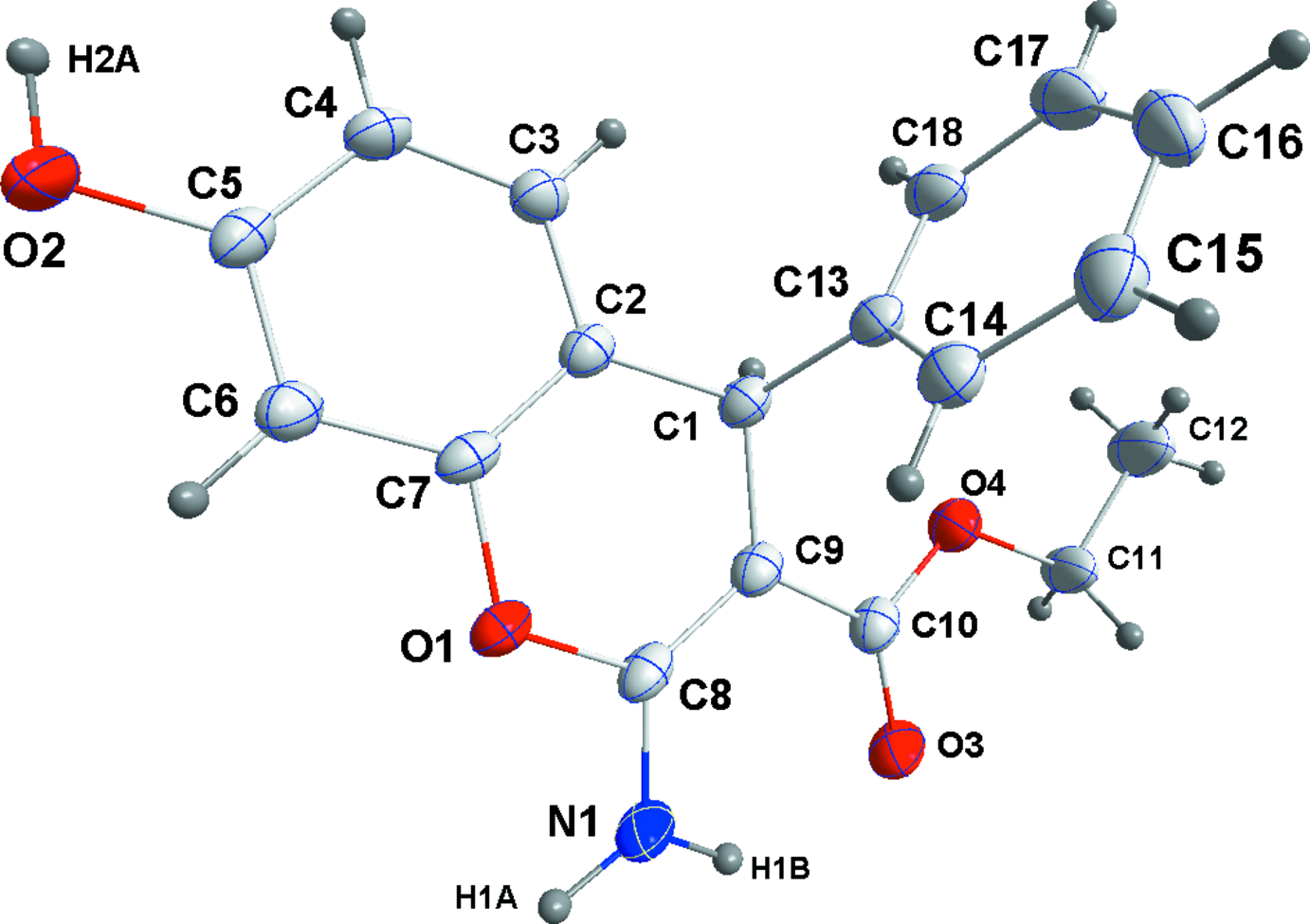

Supplement: Supplementary file 4 [file e-71-0o519-fig1.tif]

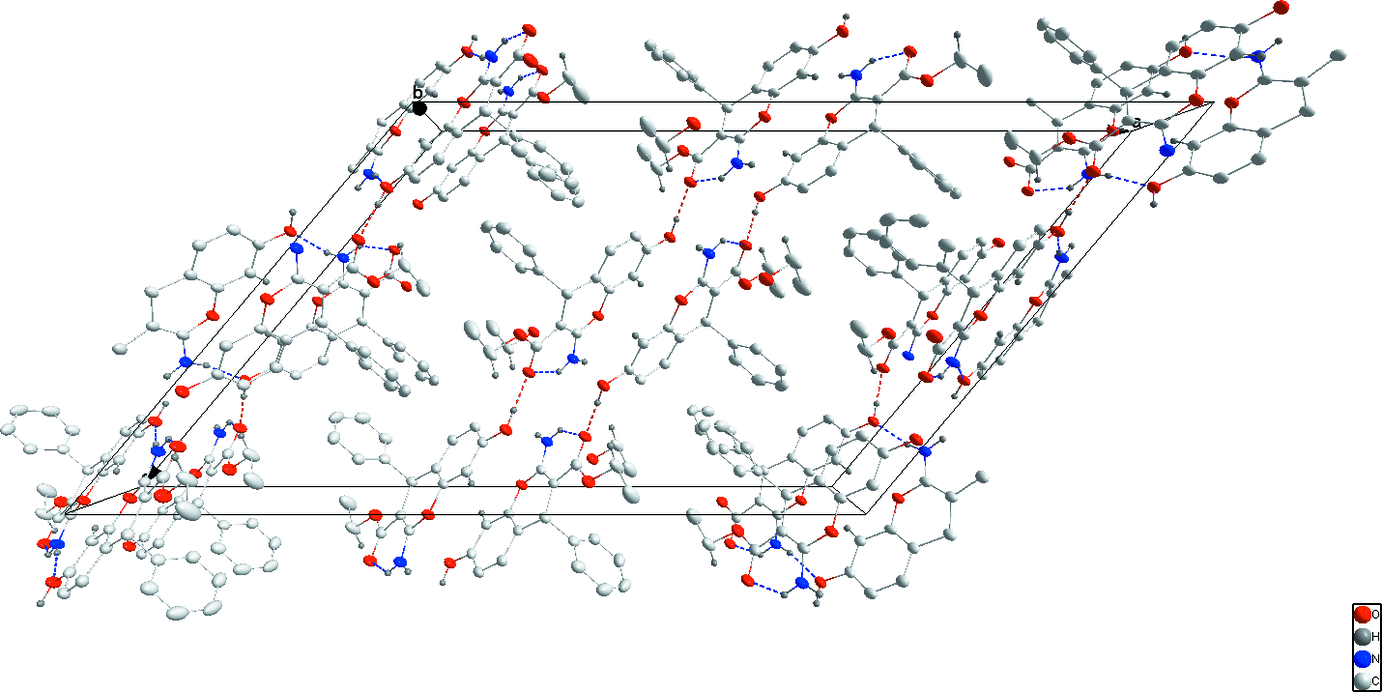

Supplement: Supplementary file 5 [file e-71-0o519-fig2.tif]
